# Biodegradable Levan/Chitosan Composite Films: Development and Application in Beef Filet Packaging

**DOI:** 10.3390/foods14122133

**Published:** 2025-06-18

**Authors:** Anissa Haddar, Emna Sellami, Oumayma Bouazizi, Assaad Sila, Ali Bougatef

**Affiliations:** 1 Laboratory of Plants Improvement and Valorization of Agroressources, National School of Engineering of Sfax, University of Sfax, Sfax 3000, Tunisia; anissa.haddar@isbs.usf.tn (A.H.); sellamiemna580@gmail.com (E.S.); oumaima.bouazizi@enis.tn (O.B.); assaadsila@gmail.com (A.S.); 2 High Institute of Biotechnology, University of Sfax, Sfax 3000, Tunisia; 3 Faculty of Sciences, University of Gafsa, Gafsa 2112, Tunisia

**Keywords:** levan, chitosan, biodegradable film, water solubility, antibacterial activity

## Abstract

This study developed biodegradable levan–chitosan composite films for beef filet packaging, optimizing their functional properties and assessing their efficacy in food preservation. Films were prepared via solution casting using varying levan–chitosan ratios (100:0, 75:25, 50:50, 25:75, 0:100) with glycerol (30%) as a plasticizer. The L25:C75 formulation exhibited superior mechanical properties (tensile strength: 15.43 ± 0.04 MPa; elongation at break: 35.37 ± 1.12%) and high-water solubility (46.64 ± 0.37%). FTIR analysis confirmed intermolecular interactions between levan and chitosan. The films demonstrated strong antioxidant activity (91% ABTS•+ inhibition) and broad-spectrum antibacterial effects, with inhibition zones of 18 ± 0.30 mm against *E. coli* and 16 ± 0.20 mm against *S. aureus*. When applied to beef filets stored at 4 °C for 7 days, the L25:C75 films reduced total viable bacterial counts by 1.8 log CFU/g compared to LDPE packaging. Biodegradability tests revealed a 68% degradation rate for high-levan films (L75:C25) after 14 days in soil. These results highlight the potential of levan–chitosan films as sustainable, active packaging materials to extend food shelf life while addressing environmental concerns.

## 1. Introduction

Food packaging plays a key role in maintaining food quality and extending shelf life [1]. While petroleum-based polymers offer good performance, their poor biodegradability causes significant environmental pollution [2]. On the other hand, paper-based packaging is safe but lacks durability and water resistance. To tackle these issues, researchers have explored biodegradable films as eco-friendly alternatives [3]. Edible packaging materials, including polysaccharides, proteins, lipids, and their blends, have emerged as promising options for sustainable food packaging. Many biopolymers derived from seafood waste show great potential for food packaging applications. Chitin, extracted from the exoskeletons of arthropods such as lobster shells, serves as a precursor to chitosan, its deacetylated, soluble, and semi-crystalline cationic form [4,5,6]. Gelatin, sourced from marine waste such as fish skin and scales, is known for forming transparent and flexible biodegradable films [6], while alginate, another biocompatible and biodegradable biopolymer, can be extracted from seaweed waste [7,8,9,10]. Chitosan dissolves in diluted organic acids like acetic, citric, lactic, tartaric, and formic acids [11], and is primarily obtained from shrimp and crab, though other sources like lobsters and oysters have also been utilized [12]. Chitosan has attracted considerable interest in food and biomedical industries due to its biocompatibility, biodegradability, and non-toxicity [13]. It exhibits notable antioxidant and antimicrobial activity against both Gram-positive and Gram-negative bacteria, owing to its positive charge which interacts with the negatively charged microbial cell membranes [14]. Furthermore, chitosan’s functional groups provide excellent compatibility with other polysaccharides, including starch, cellulose, alginate, and mucilage, making it effective for coating and film-forming applications when combined with various polymers [15]. Levan is an underexplored fructose homopolysaccharide characterized by predominantly β(2→6)-glycosidic bonds with β(2→1)-linked branches and a terminal D-glucosyl residue [16]. Produced by few plant species and a diverse range of microorganisms in both short and long chains [17], levan possesses valuable properties such as biocompatibility, biodegradability, renewability, flexibility, and eco-friendliness [18,19,20]. It is increasingly sought after in the pharmaceutical industry due to its hypoglycemic, immunostimulatory, anti-diabetic, anti-inflammatory, antioxidant, cholesterol-lowering, and anticancer activities [21,22]. Beyond its biological activity, levan serves as a food additive and film-forming agent with recognized prebiotic effects [23,24,25]. However, films composed solely of levan tend to be fragile because of its complex, compact structure; therefore, it is often combined with plasticizers or gelling agents to improve film stability. Various levan-based films have been developed by blending it with materials such as cassava starch [20,26], chitosan/polyethylene oxide (PEO) [27], pullulan/chitosan/ε-polylysine [28], sodium montmorillonite clay [29], aminoalkyl methacrylate copolymer RS [30], zinc (II) phthalocyanine [31], bentonite clay [32], ostrich eggshell [33], and chia seed mucilage [34]. Additionally, levan is utilized in the fabrication of nanocarriers, including nanofilms, nanocomposites, nanogels, nanofibers, and nanoparticles, which find applications across food, nutrition, cosmetology, pharmacy, and medicine [35,36]. Plasticizers are essential additives in the formulation of biodegradable films, as they enhance the material’s mechanical properties by increasing polymer chain mobility. These low molecular weight compounds, often in resin or liquid form, reduce the polymer’s second-order transition temperature by bonding with polymer chains, which decreases intermolecular forces and creates space between chains, resulting in a softer, more flexible, and more durable material [37,38,39,40]. Among plasticizers, polyols such as glycerol, sorbitol, and glucose are commonly used for biodegradable polymers. When combined with chitosan, glycerol and glucose improve its mechanical properties, whereas sorbitol does not have the same effect [41,42,43,44,45]. For instance, Razali et al. [46] showed that the optimal use of polyols like glycerol or polyethylene glycol enhances the mechanical strength, tensile strength, flexibility, and stability of gellan gum films intended for edible packaging; however, an excess of glycerol concentration can negatively affect tensile strength and thermal stability.

This study aimed to develop levan–chitosan composite films for use as edible packaging. The films were analyzed using Fourier transform infrared (FTIR) spectroscopy, mechanical testing, and measurements of thickness, moisture content, wettability, water solubility, and optical properties. Additionally, their antioxidant and antibacterial properties were evaluated.

## 2. Materials and Methods

### 2.1. Chitosan Preparation

Chitosan was extracted from shrimp waste using the method outlined by Sila et al. [47]. Deproteinization was carried out in a thermostatic Pyrex reactor (300 mL) with constant stirring. Shrimp waste (15 g) was homogenized with 30 mL of distilled water, and the pH was adjusted to 8.0. Proteins were digested using barbel proteases at 40 °C, and the reaction was stopped by heating the mixture to 90 °C for 20 min to inactivate the enzymes. The solid fraction was washed and pressed through four layers of gauze. This solid was then treated with 1.5 M HCl at a 1:10 (*w*/*v*) ratio for 6 h at 25 °C with constant stirring (150 rpm). The resulting chitin was further processed with 50% (*w*/*v*) NaOH at 80 °C for 4 h to achieve full deacetylation, converting it to chitosan, which is soluble in mildly acidic conditions. After filtration, the residue was washed with distilled water and dried in a dry heat incubator at 50 °C overnight. The final chitosan had a degree of deacetylation of approximately 85%.

### 2.2. Production and Purification of Levan from Bacillus mojavensis

Levan was produced using a culture medium with sucrose as the main carbon source. The medium composition (in g/L) was as follows: 50 sucrose, 0.4 CaCl_2_, 1.5 K_2_HPO_4_, 0.2 KH_2_PO_4_, 0.15 MgSO_4_·7H_2_O, and 2 yeast extract. The pH of the culture was adjusted to 7.0 before autoclaving, and the flasks were incubated in an orbital shaker at 150 rpm and 37 °C for 48 h. Following ethanol precipitation and dialysis, levan was purified by ethanol precipitation fractionation, as previously reported by Haddar et al. [16].

### 2.3. Edible Film Production

Edible films were prepared by casting with levan, chitosan, and glycerol as plasticizers. For each experiment, films were made with a fixed concentration of solids (3 g/100 g filmogenic solution) consisting of levan and chitosan, and a fixed glycerol concentration (30 g/100 g solids). The composite (levan/chitosan) solutions were obtained by mixing film-forming solutions of levan and chitosan at different ratios (G75:C25, G50:C50, G25:C75) and the resulting films were labeled as L75:C25, L50:C50, and L25:C75, respectively. Films prepared from levan (L100:C0) or chitosan (L0:C100) were used as controls. Chitosan powder was dissolved in 1% (*v*/*v*) acetic acid and levan powder in distilled water. Glycerol was added as plasticizer to the levan and/or chitosan solutions. The filmogenic solutions were dried at 35 °C in a ventilated oven until they reached a constant weight, which took approximately 24 h. The resulting translucent films were easily removed from the plates and equilibrated at 25 °C with a relative humidity (RH) of 58% for 48 h before testing.

### 2.4. Film Characterization

#### 2.4.1. Thickness, Moisture Content and Wettability

The film thickness was measured using a micrometer (Mitutoyo, Model ID-C112PM, Kawasaki-shi, Japan), with thickness recorded at ten random points on each sample. The water content in the film was calculated by subtracting the initial dry mass (*W_i_*) from the mass after drying (*W_f_*).

Wettability was assessed by measuring the static water contact angle (WCA) on each film’s surface at room temperature using a Dataphysics OCA 20 instrument, DataPhysics Instruments GmbH, Filderstadt, Germany. A 3 μL sessile drop of ultrapure water was placed on the film strip (10 mm × 60 mm) using a micro-syringe. The contact time between the water droplet and the film was 30 s. The WCA was determined using the Laplace–Young method, which correlates the pressure difference between water and air with surface shape and tension. Ten droplet images were obtained for each film surface.

#### 2.4.2. Water Solubility

Film samples (3 cm × 2 cm) were weighed and placed in 50 mL centrifuge tubes containing 10 mL of distilled water with 0.1% (*w*/*v*) sodium azide. The tubes were stirred at room temperature for 24 h. Afterward, the remaining undissolved film was removed by centrifugation at 3000× *g* for 10 min at 25 °C and then dried at 105 °C for 24 h. The test was performed in triplicate. Film solubility was determined using the following equation (Equation (1)):

(1) $$WS(\%)=\left(\frac{W_{i}–W_{f}}{W_{i}}\right)\times 100$$

where *W_i_* was the initial weight expressed as dry matter and *W_f_* was the weight of the undissolved film residue.

#### 2.4.3. Opacity Measurement

The opacity of the films was assessed following the method described by Liu et al. [48]. Rectangular film samples were prepared, and their absorbance at 600 nm was measured using a UV spectrophotometer (UV mini 188 1240, UV/VIS spectrophotometer, SHIMDZU, Beijing, China) with air as the reference. Film opacity was calculated using the following equation (Equation (2)):

(2) $$Opacity=\left(\frac{Abs600}{T}\right)$$

where Abs600 represents the absorbance at 600 nm, and T is the film thickness (mm).

#### 2.4.4. Color

The color of the film samples was determined using a ColorFlex spectrocolorimeter (Hunter Associates Laboratory, Inc., Reston, VA, USA). Color of the film was expressed as L* (lightness/brightness), a* (redness/greenness), and b* (yellowness/blueness) values. Total difference in color (ΔE*) was calculated according to the following equation (Equation (3)):

ΔE* = (ΔL*)^2^ + (Δa*)^2^ + (Δb*)^2^ (3)

where ΔL*, Δa*, and Δb* are the differences between the corresponding color parameter of the sample and that of white standard (L* = 92.84, a* = −1.25 and b* = 0.49).

#### 2.4.5. Fourier Transform Infrared (FT-IR) Analysis of the Films

Fourier Transform Infrared (FT-IR) analysis of levan, chitosan, and the films made from them were performed using an FT-IR spectrometer (Perkin Elmer Spectrum BX FT-IR, Shelton, CT, USA) at room temperature. The spectra were recorded over a wavelength range of 500–4000 cm^−1^.

#### 2.4.6. Mechanical Properties

The mechanical properties of the films were tested using a Microelectronics Universal Testing Instrument (Model TA.HD Plus, Stable Micro Systems, Godalming, UK) with a 300 N load cell, following the ISO 527-3 standard method [49]. To determine tensile strength (TS, MPa) and elongation at break (EAB, %), the films were cut into dumbbell-shaped specimens (2.5 cm × 8 cm) using a precision cutter (Thwing-Albert JDC Precision Sample Cutter, West Berlin, NJ, USA). All samples were conditioned at 25 ± 1 °C and 50 ± 1% relative humidity (RH) for two weeks before testing. The samples were then placed vertically in the testing machine’s extension grips and stretched uniaxially at a crosshead speed of 50 mm/min until they broke. Measurements were performed at room temperature (25 ± 1 °C) and 40 % RH, with six samples used for each composite film formulation.

#### 2.4.7. Antioxidant Activity of Films

The antioxidant activity of each developed film was assessed using ABTS method [50]. A 7 mM ABTS solution was prepared with 2.45 mM potassium persulfate and kept in the dark at room temperature for 16 h to allow the formation of ABTS•+. The ABTS•+ solution was then diluted in ethanol (1:80), and the concentration was adjusted to achieve an absorbance between 0.700 and 0.800 at 734 nm using a spectrophotometer (Jenway 6405 UV/Vis, Staffordshire, UK). Three replicates of each film (15 mm × 15 mm) were immersed in 1.5 mL of the ABTS•+ solution and left to react under dark conditions at room temperature with orbital stirring at 80 rpm. An ABTS•+ solution without film served as the blank. The absorbance at 734 nm was measured in triplicate over a period of 72 h. The antioxidant activity was calculated as the ABTS•+ inhibition percentage using the following equation (Equation (4)):

(4) $$Inhibition\left(\%\right)=100\times \frac{\left(Ab-Af\right)}{Ab}$$

where *Ab* and *Af* represent the absorbance values of the ABTS•+ solution incubated without and with the film, respectively.

#### 2.4.8. Antibacterial Activity of Films

The antibacterial activity of the composite films was evaluated using the disk agar diffusion method against nine foodborne pathogenic bacteria. The microorganisms tested included four Gram-positive strains (Staphylococcus aureus ATCC 25923, Micrococcus luteus ATCC 4698, *Listeria monocytogenes* ATCC 43251, *Enterococcus faecalis* ATCC 29212, and *Bacillus subtilis* ATCC 6633) and four Gram-negative strains (*Escherichia coli* ATCC 25922, *Klebsiella pneumoniae* ATCC 13883, *Salmonella enterica* ATCC 43972, and *Pseudomonas aeruginosa* ATCC 27853). A 100 μL culture suspension of each microorganism, containing approximately 10^6^ colony-forming units (CFU), was spread over Luria–Bertani (LB) agar plates. The films (1 cm × 1 cm) were sterilized under UV light for 30 min before being placed on the agar surface. The plates were then incubated at 37 °C for 24 h. Antagonistic zones were identified by the presence of clear areas below or around the films, indicating positive antibacterial activity.

### 2.5. Preparation of Beef Filets

Fresh beef filets were procured within two hours post-slaughter from a local abattoir in Sfax, Tunisia, and transported to the laboratory under refrigerated conditions (4 °C). Upon arrival, the meat was aseptically trimmed to remove visible fat and connective tissue, then cut into uniform pieces measuring 5 × 5 × 2 cm^3^ and weighing 25 ± 0.5 g. The samples were randomly divided into two groups: (1) control samples wrapped in commercial low-density polyethylene (LDPE), and (2) test samples wrapped in levan–chitosan (L25:C75) composite films, previously sterilized by UV irradiation for 30 min. Each piece was individually placed in sterile Petri dishes and fully wrapped with the respective packaging material. All samples were stored at 4 °C and subjected to microbiological analysis in triplicate on days 1, 3, 5, and 7 of refrigerated storage.

### 2.6. Microbiological Analysis of Beef Filets During Storage

Microbiological examinations were conducted on the beef filets throughout the storage period. A sterile stomacher filter bag containing 25 g of beef filets was used, and 225 mL of sterile peptone-water solution was aseptically added. The mixture was homogenized for 3 min using a BA6021 stomacher 400 (Seward, Worthing, UK). Serial dilutions were then prepared, and 100 µL from each dilution was spread onto the surface of sterile nutrient agar plates. Total aerobic counts were enumerated on Plate Count Agar (PCA) and incubated at 30 °C for 48 h. Coliform enumeration was conducted on Violet Red Bile Lactose agar (VRBL), with incubation for 24 h at either 37 °C for total coliforms or 44 °C for fecal coliforms. Psychrophilic bacteria were assessed using PCA after incubation at 4 °C for 5 days.

### 2.7. Biodegradability

The biodegradability of the developed films was evaluated using a soil burial degradation test, following the methodology of Alqahtani et al. [51], with slight modifications. First, the films were cut into 4 × 4 cm^2^ pieces and dried in an oven at 60 °C until reaching a constant weight. The samples were then buried at a depth of 4 cm in natural soil for 12 days. To maintain soil moisture, 20 mL of water was sprayed daily.

Every two days, the samples were retrieved, thoroughly washed with distilled water, and dried again at 60 °C until a constant weight was achieved. The biodegradability was determined as the percentage of weight loss using the following equation (Equation (5)):

(5) $$Biodegradability\left(\%\right)=\frac{\left(W0-W1\right)}{W0}\times 100$$

where *W*0 represents the initial dry weight of the film sample (g) and *W*1 is its dry weight after 14 days (g).

### 2.8. Statistical Study

The data were expressed as a mean followed by standard deviation (SD) with GraphPad Prism 9.02 for Windows (GraphPad, San Diego, CA, USA). Results were analyzed using one-way analysis of variance (ANOVA) followed by the Tukey test. Differences were considered significant when *p* < 0.05.

## 3. Results

### 3.1. Levan and Chitosan-Based Films

**Figure 1:**
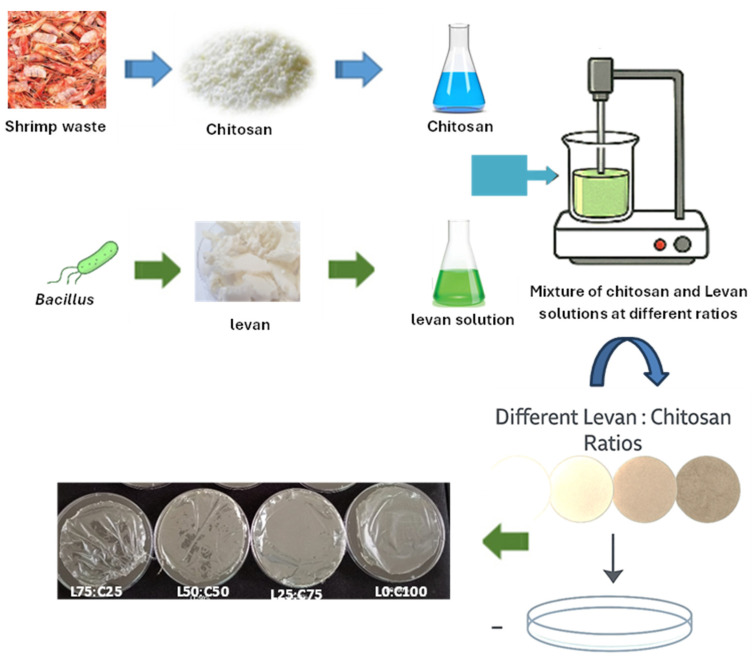
Preparation process of the levan–chitosan powder films. L0:C100: chitosan-control film; L25:C75: chitosan–levan film with 25% levan powder; L50:C50: chitosan–levan film with 50% levan powder; L75:C25: chitosan–levan film with 75% levan powder.

**Figure 2:**
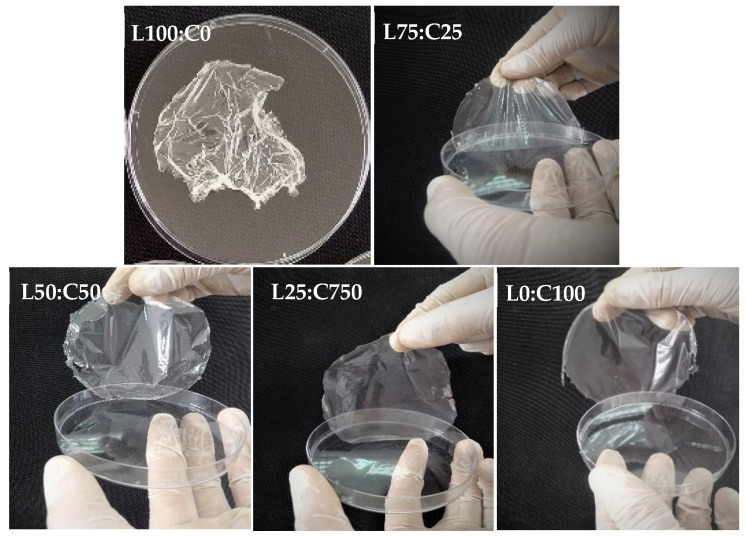
Visual appearance of films.

The incorporation of levan, a biocompatible and highly branched fructan [52], into the chitosan matrix enhanced the mechanical flexibility and biodegradability of the resulting films. These effects may be attributed to molecular interactions between levan and chitosan chains, as supported by FTIR and tensile strength results. Chitosan, widely recognized for its excellent film-forming ability, and antioxidant and antimicrobial properties [53], exhibits some limitations, such as poor thermal stability and limited water solubility. Blending it with levan helped overcome these drawbacks. This synergy is evident in the combined formulation, which showed a favorable balance of structural integrity, antioxidant capacity, and biodegradability. These findings support the idea that polymer blending is an effective approach for enhancing the functional performance of chitosan-based films, as demonstrated in previous studies [54,55]. Levan produced by *B. mojavensis* was easily dissolved in aqueous filmogenic solution to obtain the films. In film formulation, levan was blended with a chitosan solution, resulting in rapid and complete dissolution. Glycerol (30%) was used as a plasticizer to enhance the film flexibility and decrease brittleness. The levan content, starting at an initial concentration of 3%, was gradually reduced as the chitosan concentration increased (Table 1). The film made from levan alone (without chitosan) was too sticky to be removed from the Petri dish (Figure 1), a result consistent with previous studies on levan [32,56]. Films made from a levan–chitosan blend were transparent, odorless, and stretchable. The visual appearance of films made with different levan and chitosan concentrations was compared and documented. The film (L75:C25) prepared from the mixture of 2.25 g levan and 0.75 g chitosane was found to be not easily lifted from the Petri dish, and quite flexible. Interestingly, the films L50:C50 and L25:C75 were stable and flexible. They appeared uniformly smooth and clear, without visible defects like bubbles or cracks, and could be easily handled. All the films, except the one prepared from pure levan (without chitosan), were taken forward for further characterization (Figure 2).

### 3.2. Film Characterization

#### 3.2.1. FTIR Analysis

**Figure 3:**
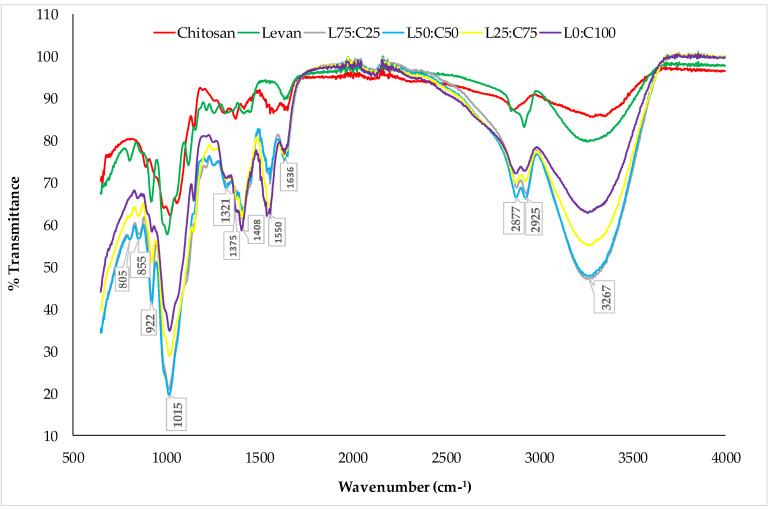
FT-IR spectra of films and their composites.

FTIR spectroscopy was performed to identify the functional groups of the compounds and to examine the interaction between the polysaccharides and plasticizers. The FTIR spectra of the polymers, levan and chitosan, as well as the films made from chitosan and levan–chitosan blends, were recorded (Figure 3).

The FTIR analysis of chitosan exhibited a stretching vibration peak of the O–H bond at 3371 cm^−1^. Additionally, absorption bands at 1576 cm^−1^ and 1418 cm^−1^ were associated with amides I and II, respectively [57]. The characteristic bands at 1036 cm^−1^ and 894 cm^−1^ corresponded to the stretching vibrations of C–N and C–O bonds in the chitosan structure [58]. Similarly, the FTIR spectrum of levan displayed a broad absorption band at 3268 cm^−1^, attributed to the stretching vibration of the O–H bond [59]. The bands around 2920 cm^−1^ and 1190 cm^−1^ were associated with C–H stretching and the O–H vibration of water, respectively [60,61]. Furthermore, the characteristic absorption bands at approximately 892 cm^−1^ and 807 cm^−1^ were assigned to the furanose ring of levan [62]. The FTIR spectra of levan/chitosan blend films confirmed the presence of characteristic peaks from both chitosan and levan, indicating the successful fabrication of the composite films. Prominent absorption bands were observed at 3267 cm^−1^ and 2925 cm^−1^, corresponding to O–H and C–H stretching vibrations, respectively [15,20,56]. Additionally, the amide-related absorption bands of chitosan appeared at 1550 cm^−1^ and 1408 cm^−1^. The characteristic absorption bands of levan’s furanose ring were also detected in the spectra, further confirming the structural integration of both biopolymers in the blend films.

#### 3.2.2. Thickness, Moisture Content, Wettability, Water Solubility, and Optical Properties

Table 2 shows the thickness, moisture content, wettability, solubility, and optical properties in the developed films. The film thickness remained relatively constant across different formulations, suggesting stable interactions between levan and chitosan. This consistent thickness indicates that the blending of levan and chitosan results in uniform film formation. Such stability in thickness is indicative of homogenous dispersion and good compatibility between the two biopolymers. The moisture content of the films gradually decreases as the proportion of chitosan increases. Films with a higher amount of levan (L75:C25) show higher moisture content (25.53%), while those composed entirely of chitosan (L0:C100) have the lowest moisture content (21.87%). This can be attributed to the hydrophilic nature of levan, which attracts and retains moisture.

Contact angle measurements were conducted to assess the impact of chitosan addition in levan coating films, with the results shown in Table 2. For films L75:C25, L50:C50, and L25:C75, the contact angles were found to be 62.5 ± 0.6°, 63.9 ± 1.2°, and 65.3 ± 0.4°, respectively. The contact angle of the chitosan film was recorded as 67.7 ± 0.2°. These results indicate that as the chitosan concentration increases, hydrogen bonding also increases, making the films less hydrophilic.

Water solubility is a key characteristic of biodegradable films, as it indicates the film’s resistance to water. Compared to the chitosan film (L0:C100), the films made from a combination of levan and chitosan exhibited higher water solubility (Table 2). Levan is a water-soluble polysaccharide; it contributes to higher solubility of the films when present in larger amounts. According to Mantovan et al. [20], the incorporation of levan, a water-soluble polysaccharide, into starch blends led to a proportional increase in film solubility. Similarly, Agarwal et al. [56] reported that the film made from a blend of levan and gellan gum exhibited higher water solubility compared to the gellan gum film. Bersaneti et al. [63] found that the solubility of starch films increased when fructooligosaccharides were incorporated. These oligosaccharides, which have prebiotic properties, share similar bonding characteristics with levan. The findings suggest that the levan–chitosan mixture provides the film material with relatively higher solubility. This increased solubility indicates a lower water resistance. Additionally, films with high water solubility are suitable for use as edible films, which dissolve readily [64].

All the films exhibited a consistent visual appearance, free from cracks or defects. The color parameters of the levan/chitosan blend films indicate variations in lightness (L*), redness/greenness (a*), and yellowness/blueness (b*) depending on the chitosan content. As the proportion of chitosan increases, the L* value also increases, indicating a lighter appearance, with values ranging from 86.33 ± 0.21 for L75:C25 to 90.35 ± 0.25 for L0:C100. The a* values, representing the red–green axis, decrease as chitosan content rises, shifting from 2.03 ± 0.03 for L75:C25 to 0.38 ± 0.05 for L0:C100, suggesting a reduction in redness. Similarly, the b* values, which indicate the yellow–blue axis, increase with higher chitosan concentrations, ranging from 10.70 ± 0.05 for L75:C25 to 14.27 ± 0.05 for L0:C100, reflecting an increase in yellowness. These findings demonstrate that chitosan contributes to a lighter and more yellow-toned film, while levan imparts a slightly darker and redder hue. Color is a key attribute that can influence consumer perception and acceptance, especially for applications in the food and packaging industries where visual appeal is important. A consistent and desirable color can enhance the marketability of the product. Additionally, color measurements can provide insights into the chemical interactions and compatibility between the components of the film.

Opacity serves as a dual function in food packaging films by enhancing visual clarity while providing partial protection against UV radiation. In this study, the opacity of chitosan–levan films showed a slight increase with higher levan content: L75:C25 (19.2%), L50:C50 (18.7%), L25:C75 (18.25%), and L0:C100 (17.5%). This trend suggests that the incorporation of levan marginally elevates film opacity, likely due to light scattering within its polysaccharide matrix rather than direct UV absorption. As a result, although chitosan–levan films offer limited UV protection through increased opacity, their effectiveness as UV barriers remains relatively low.

Sample L25:C75 has a thickness of 133 ± 0.04 μm, comparable to other films, demonstrating homogeneous manufacturing. Its low moisture content (23 ± 0.36%) suggests better resistance to water absorption. The high contact angle (65.3 ± 0.4°) reveals increased hydrophobicity, related to a reduced levan content. With a solubility of 46.64 ± 0.37%, one of the lowest, this sample confirms the effect on solubility reduction. Its intermediate opacity (18.25 ± 1.5%) indicates a good compromise between transparency and opacity.

#### 3.2.3. Mechanical Properties of the Films

The mechanical properties are crucial for determining the suitability of films for various applications. Strong mechanical resistance and flexibility are key attributes that ensure the protection of packaged products from potential damage [65]. The mechanical behavior of the blend films is characterized by parameters such as elastic modulus, elongation at break, and tensile strength, as illustrated in Table 3. The inclusion of chitosan led to a notable increase in tensile strength compared to the film with the highest levan proportion (L75:C25). The formulation with the highest proportion of chitosan (L0:C100) exhibited the highest tensile strength (17.01 ± 0.03 MPa). However, the levan–chitosan blend with the proportion of L25:C75 showed a high tensile strength of 15.43 ± 0.04 MPa, which is notable among the blended films. The elongation at break (EAB) of the levan–chitosan blended films, i.e., the maximum distance that films can stretch before breaking, exhibited a substantial decrease in elongation from 99.97 ± 1.02% to 35.37 ± 1.12% for the films with compositions L75:C25 and L25:C75, respectively. Moreover, Young’s modulus of the levan–chitosan films, i.e., the maximum force that a film can sustain before deforming considerably, increased from 0.34 MPa to 87.85 MPa for films containing 25% and 75% chitosane, respectively. Chen et al. [29] found that pure levan films prepared by casting exhibited poor mechanical properties, which they attributed to levan’s highly branched and compact structure, limiting intermolecular entanglement. Similarly, Barone and Medynets [62] reported that extruded levan films with glycerol as a plasticizer were rigid and prone to cracking when glycerol concentrations were below 15%. In contrast, this study found that using levan–chitosan–glycerol blends resulted in films with enhanced mechanical properties. These improvements suggest that the developed levan–chitosan films are well-suited for wrapping and packaging applications.

Sample L25:C75 displays a tensile strength of 15.43 ± 0.04 MPa, significantly higher than that of the other formulations, highlighting the reinforcing effect of levan on the mechanical properties. Its elongation at break reaches 35.37 ± 1.12%, lower than L75:C25, but revealing acceptable flexibility despite increased rigidity. The high Young’s modulus (87.85 ± 0.02 MPa) also confirms the increase in rigidity due to the majority presence of levan.

#### 3.2.4. Antioxidant Activity of Films

**Figure 4:**
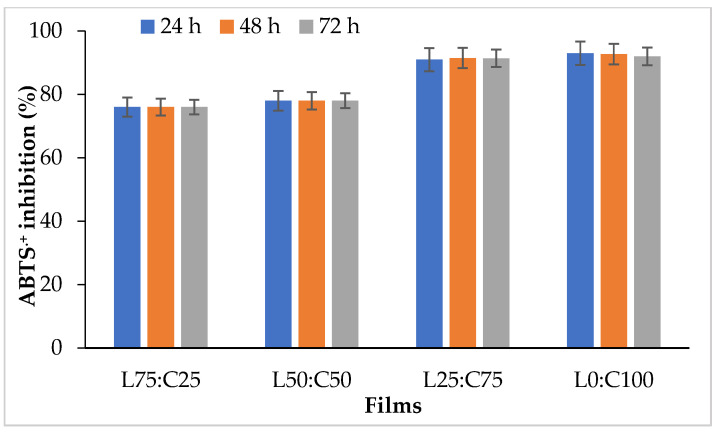
Antioxidant activity (by ABTS•+ inhibition) of levan–chitosan films after 24, 48 and 72 h of incubation.

The antioxidant activity of active films is essential for assessing their effectiveness, as it indicates their ability to prevent or delay the oxidation of treated products [66,67,68]. Several studies have highlighted that antioxidant packaging is a highly sought-after category of active packaging due to its potential to extend food shelf life. It helps prevent oxidative stress caused by free radicals and UV radiation, which are linked to the development of various human diseases [69,70]. The antioxidant activity of edible levan–chitosan films was evaluated using a free radical-scavenging activity assay (ABTS•+ inhibition). As shown in Figure 4, chitosan increased the ABTS•+ inhibition percentage in composite films. Films with a levan–chitosan ratio of 75:25 maintained stable antioxidant activity after 72 h of incubation. Notably, the films with a high chitosan content (L25:C75) exhibited strong antioxidant activity, reaching 91% over the same period. The films prepared from chitosan (devoid of levan) demonstrated high antioxidant activity, with ABTS•+ inhibition values of 93%, 92.75%, and 92% at 24, 48, and 72 h, respectively. These findings suggest that chitosan enhances the antioxidant properties of the films, likely due to its inherent antioxidant activity. This improved antioxidant activity is essential for active packaging applications as it helps extend the shelf life of food products by preventing oxidative stress. Aranaz et al. [71] reported that chitosan exhibits significant scavenging capability against various radical compounds, achieving antioxidative results comparable to those of well-known antioxidants. In this study, the radical scavenging activity of levan–chitosan films can be attributed to chitosan’s ability to supply hydrogen atoms, which serve as effective antioxidants. Additionally, the high antioxidant activity of chitosan films is due to the significant free radical scavenging ability of the free amino groups at the C-2 position of the chitosan chains. Jridi et al. [72] have reported that gelatin/chitosan composite films can function as electron or hydrogen donors, allowing them to neutralize free radicals by converting them into more stable products, thereby halting radical chain reactions. Hajji et al. [73] demonstrated that chitosan-gelatin films enriched with crustacean protein hydrolysates exhibit notable antioxidant activity. To enhance the antioxidant properties of chitosan, natural active compounds like essential oils and polyphenols have been incorporated into the chitosan matrix through physical mixing to create composite films [74]. The antioxidant capacity of active chitosan films enhanced with various spice extracts, including thyme, clove, prickly ash, fennel, geranium, and cinnamon, has been studied [75].

#### 3.2.5. Antibacterial Properties of Films

The antimicrobial activity of the films is a crucial aspect of their functional properties. Factors such as the degree of deacetylation, molecular weight, film-forming conditions, pH, and temperature influence chitosan’s antibacterial effectiveness [76,77]. Previous studies have demonstrated that chitosan can inhibit the growth of a wide range of fungi and bacteria, showing greater effectiveness against Gram-positive bacteria compared to Gram-negative bacteria [78]. Antibacterial activity of the films was evaluated using the agar diffusion method. Table 4 shows the antibacterial activity exhibited by the inhibitory zone of film against Gram-negative bacteria (*E. coli*, *P. aeruginosa*, *K. pneumoniae*, and *S. enterica*) and Gram-positive bacteria (*S. aureus*, *B. subtilis*, *M. luteus*, *E. faecalis*, and *L. monocytogenes*). Results indicate that the chitosan films and composite films (levan/chitosan) exhibited varying degrees of antibacterial activity against the Gram-positive and Gram-negative bacteria tested. The inhibitory activity increased with the increase in chitosan content in the composite films. Numerous authors have attributed the antimicrobial properties of chitosan to its positively charged amino groups. These groups interact with the negatively charged microbial cell membranes, causing leakage of proteinaceous materials and other intracellular constituents from the microorganisms [79,80,81]. Other mechanisms include the interaction of diffused hydrolysis products with microbial DNA, resulting in the inhibition of mRNA and protein synthesis [82,83], and the chelation of metals, spore elements, and essential nutrients [84]. In their review, No et al. [85] highlighted the inherent antibacterial and antifungal properties of chitosan and its applications for improving the quality and shelf life of various foods from agricultural, poultry, and seafood origins. Because of these properties, chitosan is considered a perfect material for the development of films for food use. Gómez-Estaca et al. [86] reported that fish gelatin–chitosan films, despite the interactions between gelatin and chitosan, exhibited antimicrobial activity against *Staphylococcus aureus*, a significant cause of food poisoning. Jridi et al. [72] showed that blending gelatin and chitosan at ratios of G75/C25 or G50/C50 can enhance the physicochemical performance of composite films without altering the antimicrobial properties of chitosan. Gan et al. [28] demonstrated that levan/pullulan/chitosan edible films, enriched with ε-polylysine, exhibit antibacterial activity against *Escherichia coli* and *Staphylococcus aureus*, which are typical foodborne pathogens. Mujtaba et al. [15] investigated the effect of composite coatings on the shelf life of cherry fruits. They applied coatings made from chitosan, chia mucus, and levan to cherries and evaluated their performance for post-harvest preservation under both market and refrigeration conditions.

Sample L25:C75 demonstrates significant inhibition against all microorganisms tested, with zones of inhibition comparable to those of L0:C100, notably against *E. coli* (18 ± 0.30 mm), *P. aeruginosa* (19 ± 0.25 mm), *S. aureus* (14 ± 0.20 mm), and *B. subtilis* (16 ± 0.20 mm). These results indicate that L25:C75 retains strong antimicrobial activity despite a change in composition.

### 3.3. Microbiological Analysis of Beef Filets

Microbial contamination of foods is a significant concern due to its impact on public health and the prevalence of foodborne diseases [87]. As a result, the packaging industry is exploring solutions that offer functional properties such as specific gas barriers, antioxidant activity, and antimicrobial capabilities. These innovative packaging materials aim to extend food shelf life, allowing for longer storage periods [88]. Active packaging interacts with food to maintain its nutritional quality, prevent the growth of harmful microorganisms, and stop the migration of contaminants [74]. As shown in Table 5, microbiological analysis of beef filets packaged in active levan/chitosan films (L25:C75) and stored at 4 °C for 7 days revealed significant differences compared to those packaged in LDPE. The total viable count of beef filets wrapped in LDPE increased from 3.25 ± 0.25 log CFU/g on day 1 to 6.30 ± 0.27 log CFU/g by day 7, while those packaged in L25:C75 films showed a slower increase, reaching 4.50 ± 0.25 log CFU/g by day 7.

The count of psychrophilic bacteria increased more rapidly in beef filets packaged in LDPE, rising from 2.50 ± 0.12 to 5.64 ± 0.15 log CFU/g, compared to a slower increase from 2.35 ± 0.10 to 3.95 ± 0.28 log CFU/g in filets packaged in L25:C75 films. Coliforms, which are commonly used as indicators of food hygiene [89], also showed higher counts in LDPE-packaged filets (1.95 ± 0.15 to 3.55 ± 0.05 log CFU/g) than in those packaged in L25:C75 films (1.75 ± 0.12 to 2.90 ± 0.09 log CFU/g). Fecal coliforms followed the same trend, increasing from 1.55 ± 0.20 to 3.20 ± 0.25 log CFU/g in LDPE-packaged filets, while counts in L25:C75-packaged filets were lower, ranging from 1.36 ± 0.23 to 2.28 ± 0.25 log CFU/g. These results are consistent with findings by Alirezalu et al. [90], who demonstrated that chitosan films, with or without ε-Polylysine, are more effective than LDPE in inhibiting microbial growth in beef filets and maintaining quality. Similarly, Wang et al. [21] formulated a levan–chitosan (LE/CS) blend films, which were assessed as a packaging material for fresh pork. The results demonstrated its effectiveness in maintaining meat quality. Studies by Kakaei and Shahbazi [91] and Sun et al. [92] explored the potential of active chitosan films in preventing the growth of *L. monocytogenes* and their antioxidant properties in different fishery products.

Sample L25:C75 significantly reduced microbial growth in beef filets compared to LDPE films, with total viable counts of 4.50 ± 0.25 versus 6.30 ± 0.30 after 7 days, psychrophilic bacterial load of 3.95 ± 0.30 versus 5.64 ± 0.15, and a decrease in coliforms of approximately 0.5 to 1 log CFU/g. These results confirm the effectiveness of L25:C75 in extending food shelf life.

### 3.4. Biodegradability

**Figure 5:**
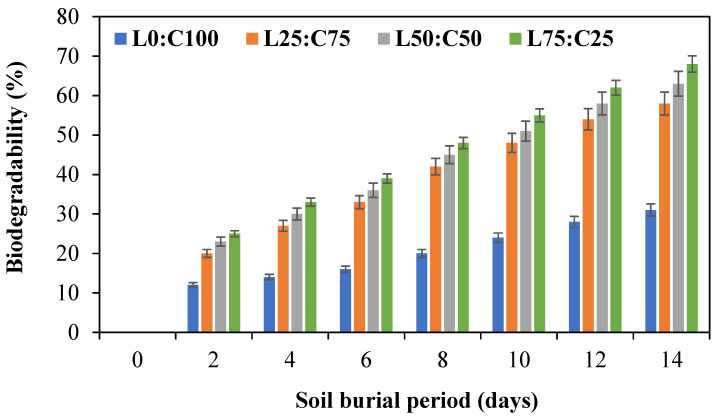
Biodegradability (%) of chitosan–levan composite films over 14 days of soil burial. L0:C100: chitosan control film; L25:C75: chitosan–levan film with 25% levan; L50:C50: chitosan–levan film with 50% levan; L75:C25: chitosan–levan film with 75% levan.

The biodegradability study of chitosan–levan films over 14 days of soil burial showed that increasing levan content significantly enhances film degradation (Figure 5). While the pure chitosan film (L0:C100) exhibited the lowest biodegradability (31%), films with higher levan ratios, particularly L75:C25, reached up to 68%. This trend suggests that levan promotes microbial accessibility and moisture absorption, accelerating biodegradation. These results align with previous findings that levan, being an amorphous and hydrophilic polysaccharide, improves the degradation rate when blended with chitosan, making such films promising for environmentally friendly applications. These findings are consistent with previous studies reporting that chitosan degrades slowly due to its semi-crystalline structure and limited enzymatic attack [92,93], while levan, a hydrophilic and amorphous fructan, enhances degradation when blended with other biopolymers [94,95]. Such blends offer promising potential for biodegradable packaging and biomedical applications.

## 4. Conclusions

This study demonstrated that combining microbial levan, produced by *Bacillus mojavensis*, with chitosan derived from shrimp waste through the solution casting method, led to composite films with improved properties. The effects of varying proportions of levan and chitosan in the blend films on their morphology, structure, mechanical performance, as well as antioxidant and antimicrobial properties, were thoroughly analyzed and discussed. The homogeneous structure and smooth texture of the composite levan/chitosan films were noted, indicating the high compatibility of these polymers. Moreover, FTIR analysis revealed intermolecular interactions between chitosan and levan. The addition of chitosan improved the mechanical performance. The levan/chitosan blend film with a ratio of L25/C75 exhibited the best mechanical properties, with a tensile strength of 15.43 MPa, indicating that chitosan enhanced the stiffness of levan films. Interestingly, the combination of levan and chitosan may enhance the antioxidant effect of composite films while preserving the antimicrobial properties of chitosan. Moreover, the results showed that levan/chitosan films were more effective in maintaining the quality of beef filets by inhibiting microbial growth, compared to LDPE films. We concluded that levan and chitosan composite films are a promising option for food packaging due to their ease of formation, excellent antimicrobial and antioxidant properties, non-toxicity, and biodegradability. These films also offer a significant extension of the shelf life of beef filet, making them a natural alternative for its preservation.

## Figures and Tables

**Table 1:** Films produced using different compositions of levan and chitosan.

S. No.	Levan (g)	Chitosan (g)	Glycerol (g)	Water (g)	Film Properties
1 (L100:C0)	3	0	0.9	96.1	The film could not be removed from the Petri dish.
2 (L75:C25)	2.25	0.75	0.9	96.1	Quite flexible and not easily lifted from the Petri dish.
3 (L50:C50)	1.5	1.5	0.9	96.1	Easily handled, flexible, transparent without bubbles or cracks.
4 (L25:C75)	0.75	2.25	0.9	96.1	Easily handled, flexible, transparent uniformly smooth, and clear.
5 (L0:C100)	0	3	0.9	96.1	Easily lifted from the Petri dish and flexible.

**Table 2:** Thickness, moisture content, wettability and solubility of levan–chitosan films.

Film Sample	Thickness (μm)	Moisture Content (%)	Water Contact Angle (°)	Opacity (%)	Solubility (%)	Color Parameters		
						L*	a*	b*
L75:C25	131 ± 0.09	25.53 ± 0.35	62.5 ± 0.6 *	19.2 ± 2 *	73.36 ± 0.25 *	86.33 ± 0.21	2.03 ± 0.03	10.70 ± 0.05
L50:C50	132 ± 0.05	24.95 ± 0.24	63.9 ± 1.2 *	18.7 ± 1.5 *	55.03 ± 0.09 *	88.54 ± 0.15	1.54 ± 0.01	11.21 ± 0.01
L25:C75	133 ± 0.04	23.05 ± 0.36 *^¥^	65.3 ± 0.4 *^¥^	18.25 ± 1.5 *^¥^	46.64 ± 0.37 *	89.45 ± 0.12 *	0.87 ± 0.02 *	12.26 ± 0.10 *
L0:C100	133 ± 0.04	21.87 ± 0.19	67.7 ± 0.2	17.5 ± 1.25	40.94 ± 0.24	90.35 ± 0.25	0.38 ± 0.05	14.27 ± 0.05

* *p* < 0.05: L75:C25, L50:C50, L25:C75 vs. L0:C100; ^¥^ *p* < 0.05: L25:C75 vs. L75:C25, L50:C50.

**Table 3:** Mechanical properties of levan–chitosan films.

Film Sample	Tensile Strength (MPa)	Elongation (%)	Young’s Modulus (MPa)
L75:C25	0.41 ± 0.06 *	99.97 ± 1.02 *	0.34 ± 0.04 *
L50:C50	2.57 ± 0.06 *	54.03 ± 3.36 *	6.00 ± 0.05 *
L25:C75	15.43 ± 0.04 *	35.37 ± 1.12 *^¥^	87.85 ± 0.02 *^¥^
L0:C100	17.01 ± 0.03	40.53 ± 2.60	114.03 ± 0.03

* *p* < 0.05: L75:C25, L50:C50, L25:C75 vs. L0:C100; ^¥^ *p* < 0.05: L25:C75 vs. L75:C25, L50:C50.

**Table 4:** Antimicrobial activity of films.

Indicator Organism		Inhibition (mm)			
		L75:C25	L50:C50	L25:C75	L0:C100
**Gram −**	*E. coli*	15 ± 0.30	17 ± 0.20 *	18 ± 0.30 *^¥^	18 ± 0.20
	*K. pneumoniea*	13 ± 0.25	15 ± 0.20	16 ± 0.40 *	16 ± 0.25
	*P. aeruginosa*	16 ± 0.30 *	18 ± 0.15 *	19 ± 0.25 *^¥^	19 ± 0.15
	*S. enterica*	14 ± 0.20	15 ± 0.25 *	15 ± 0.30	16 ± 0.20
**Gram +**	*S. aureus*	12 ± 0.25	12 ± 0.30	14 ± 0.20	14 ± 0.20
	*B. subtilis*	13 ± 0.25	14 ± 0.20	16 ± 0.20 *	16 ± 0.30
	*M luteus*	10 ± 0.3	10 ± 0.15	12 ± 0.25 *^¥^	13 ± 0.30
	*E. faecalis*	11 ± 0.25	13 ± 0.40 *	13 ± 0.30 *^¥^	14 ± 0.30
	*L. monocytogenes*	10 ± 0.40	10 ± 0.15 *	11 ± 0.25 *	11 ± 0.35

* *p* < 0.05: L75:C25, L50:C50, L25:C75 vs. L0:C100; ^¥^ *p* < 0.05: L25:C75 vs. L75:C25, L50:C50.

**Table 5:** Evaluation of microbiological counts (log CFU/g) in beef filets packaged in active levan–chitosan film (L25:C75) stored at 4 °C for 7 days.

Microbes	Packaging Films	Storage Time (Day)			
		1	3	5	7
Total viable counts (log UFC g^−1^)	LDPE films	3.25 ± 0.25	3.89 ± 0.20	4.75 ± 0.15	6.30 ± 0.30
	L25:C75 films	3.20 ± 0.15	3.50 ± 0.15	3.85 ± 0.10	4.50 ± 0.25 *
Psychrophilic bacteria (log UFC g^−1^)	LDPE films	2.50 ± 0.10	3.20 ± 0.25	4.15 ± 0.25	5.64 ± 0.15
	L25:C75 films	2.35 ± 0.10 *	2.75 ± 0.20	3.10 ± 0.20	3.95 ± 0.30 *
Total coliform (log UFC g^−1^)	LDPE films	1.95 ± 0.15	2.35 ± 0.20	3.10 ± 0.15	3.55 ± 010
	L25:C75 films	1.75 ± 0.10	2.00 ± 0.25	2.17 ± 0.15	2.90 ± 0.10 *
Fecal coliform (log UFC g^−1^)	LDPE films	1.55 ± 0.20	2.35 ± 0.25	2.95± 0.15	3.20 ± 0.25
	L25:C75 films	1.36 ± 0.25	1.75 ± 0.20	2.10 ± 0.20	2.28 ± 0.25 *

* *p* < 0.05: L25:C75 vs. LDPE.

## Data Availability

The original contributions presented in the study are included in the article, further inquiries can be directed at the corresponding author.
